# Ultrasound-Enhanced Chemiluminescence for Bioimaging

**DOI:** 10.3389/fbioe.2020.00025

**Published:** 2020-02-06

**Authors:** Duong Le, Dinesh Dhamecha, Andrea Gonsalves, Jyothi U. Menon

**Affiliations:** Department of Biomedical and Pharmaceutical Sciences, College of Pharmacy, The University of Rhode Island, Kingston, RI, United States

**Keywords:** chemiluminescence, bioluminescence, focused ultrasound, deep tissue, imaging

## Abstract

Tissue imaging has emerged as an important aspect of theragnosis. It is essential not only to evaluate the degree of the disease and thus provide appropriate treatments, but also to monitor the delivery of administered drugs and the subsequent recovery of target tissues. Several techniques including magnetic resonance imaging (MRI), computational tomography (CT), acoustic tomography (AT), biofluorescence (BF) and chemiluminescence (CL), have been developed to reconstruct three-dimensional images of tissues. While imaging has been achieved with adequate spatial resolution for shallow depths, challenges still remain for imaging deep tissues. Energy loss is usually observed when using a magnetic field or traditional ultrasound (US), which leads to a need for more powerful energy input. This may subsequently result in tissue damage. CT requires exposure to radiation and a high dose of contrast agent to be administered for imaging. The BF technique, meanwhile, is affected by strong scattering of light and autofluorescence of tissues. The CL is a more selective and sensitive method as stable luminophores are produced from physiochemical reactions, e.g. with reactive oxygen species. Development of near infrared-emitting luminophores also bring potential for application of CL in deep tissues and whole animal studies. However, traditional CL imaging requires an enhancer to increase the intensity of low-level light emissions, while reducing the scattering of emitted light through turbid tissue environment. There has been interest in the use of focused ultrasound (FUS), which can allow acoustic waves to propagate within tissues and modulate chemiluminescence signals. While light scattering is decreased, the spatial resolution is increased with the assistance of US. In this review, chemiluminescence detection in deep tissues with assistance of FUS will be highlighted to discuss its potential in deep tissue imaging.

## Introduction

Imaging has become an essential component of biomedical research and patient treatment. There has been tremendous improvement in imaging techniques and their application in last 30 years. These imaging tools help clinicians not only to diagnose diseases but also to visualize the expression of the reaction, and interactions within the human body (Weissleder and Pittet, 2008). *In vivo* molecular imaging has significantly revolutionized modern medical diagnostics. In order to evaluate the complex nature of tissues/organs, there is a need for advanced and versatile imaging techniques which are not only capable of analyzing the structure and morphology of tissues/organs but can also efficiently monitor the functions and molecular reactions in the cells (Nam et al., 2014). Each of the imaging modalities available today work on different principles and methods, and the outcome are largely variable depending on interfaces, samples and the imaging technique used (Nam et al., 2014). Therefore, based on the experiments and the clinical application, the most appropriate imaging technique must be carefully chosen from among the range of available methods.

Tissue imaging can be done using spectroscopic signal detection techniques such as magnetic resonance imaging (MRI), computational tomography (CT), acoustic tomography (AT), biofluorescence (BF), and chemiluminescence (CL). Based on the unique principle of each technique, imaging methods have their exclusive range of applications. In most imaging methods, the physical interaction of X-rays, radiofrequencies or sound waves with the target/imaged object (tissues or organs) results in a change in the energy, which is transmitted to form an image. Based on the source and intensity of the energy, the various imaging modalities differ in their specific properties such as resolution, exogenous and endogenous contrast component, penetration depth, cost, and safety (Pysz et al., 2010; Appel et al., 2013). Due to the high energy source, CT and MRI have the best imaging depth and resolution when compared to luminescence and AT. MRI uses radio frequencies coupled with strong magnetic field as the source of energy, which rebounds off the body fat and water molecules, and the transmitted energy is detected and translated into an image. Hence, it is generally used for imaging of soft tissues like brain, wrists, heart and blood vessels (Miwa and Otsuka, 2017). In comparison to CT, MRI has two disadvantages, namely loud machine noises during imaging and longer imaging time (Weissleder and Pittet, 2008). CT uses X-ray energy to image the target tissue and is quick, painless and non-invasive. It is generally used to image bone fractures, tumors progression and internal bleeding (Pysz et al., 2010). However, one of the constraints of CT imaging is the use of radiation and the generation of less detailed images of soft tissues when compared to MRI (Nam et al., 2014). AT has evolved as a hybrid imaging method which can possibly overcome some of the disadvantages of MRI and CT. AT imaging is based on the acoustic wave signals which are generated when the absorbed optical energy is converted to acoustic energy. These waves scatter less than the optical waves in tissue, leading to generation of high-resolution images of deeper tissues. This imaging technique has several advantages. For example, in comparison with CT, it uses non-X-ray laser energy source for imaging, and in comparison to MRI, it is less expensive. However, it faces some disadvantages like poor deep tissue imaging and imaging speed (Xia et al., 2014). Luminescent imaging is another method of imaging. Bioluminescence (BL) is excellent for molecular level imaging without using an external contrast agent, and it has the capacity for real time imaging (Zhang et al., 2006; Weissleder and Pittet, 2008). BL is a unique optical imaging method in that it depends on an internal biological light source (based on a reaction) unlike other imaging systems which require external energy source. Luciferase are a group of enzymes commonly used in BL imaging as they can emit light in the presence of oxygen and a substrate (typically luciferin) (Contag and Ross, 2002). The released light generated from the live cells is assessed by a photon detector with high sensitivity (Weissleder and Pittet, 2008). Similarly, CL (first generation), without the need of external light sources, advances luminescent imaging further by using luminescent enhancers listed and described in detail in Table 1. CL avoids the need for the enzyme luciferase for imaging, thus circumventing the need for genetic modification to produce BL for imaging (Lippert, 2017). CL also allows imaging of whole tissues. Recently, ultrasound (US) modulated fluorescence and US switchable fluorescence (UF) have advanced deep tissue imaging. Although these techniques are new and still under investigation, they are supposed to have advantage over CT and MRI in terms of deep tissue imaging. The key element in these types of imaging is the design of the external contrast agent, which determines the success of the imaging (Pei and Wei, 2019). With the exponential increase in research into the medical applications of US, second generation CL, i.e., ultrasound-enhanced chemiluminescence (UECL), has been developed. In UECL, CL’s limitations in deep tissue imaging is attenuated. The attributes of CL and UECL is discussed in detail below.

**TABLE 1:** Summary of CL materials.

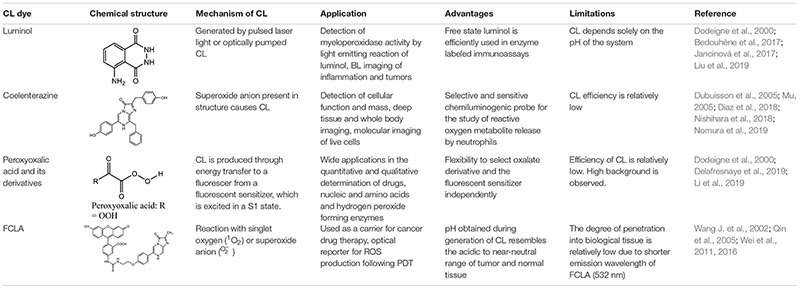

## First Generation of Chemiluminescence and Bioluminescence

Luminescence in general is defined as the emission of visible light without increase in the temperature. Luminescence emerging from a chemical reaction is known as CL. One form of CL is bioluminescence, which is basically the production and release of light by a living organism (Créton and Jaffe, 2001). BL and CL are the results of a chemical reaction in which a product at an electronically excited state returns to the ground state by emitting a photon, which is seen as light. This BL and CL light lasts for few seconds as the reaction is very fast and continues for a very short time frame. However, literature reveals that certain suitable supplementary chemicals can modify the emission kinetics in the range of 10 s to 30 min (Aslan and Geddes, 2009). This leads to the improvement in the analytical signal output with significant reproducibility (Roda et al., 2003a).

**FIGURE 1:**
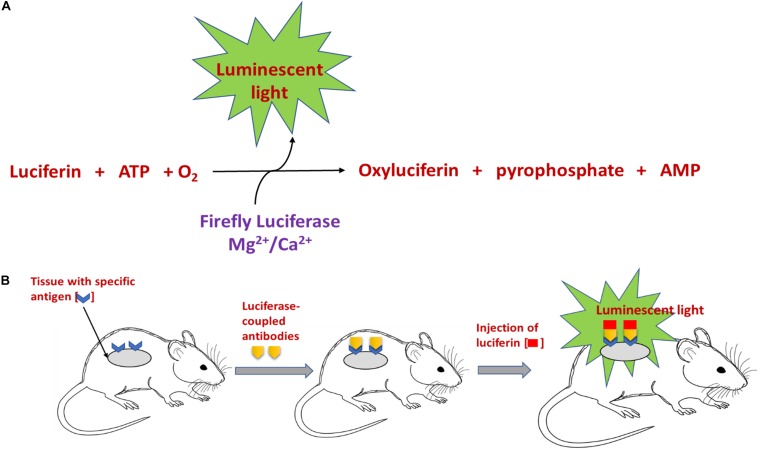
Schematic representation of **(A)** the mechanism involved in luciferin-mediated luminescence reaction, and **(B)** *in vivo* bioluminescence imaging using an antibody coupled luciferase-luciferin reaction.

Bioluminescence is observed in vertebrates and invertebrates (fireflies) and in some microorganisms like fungi and bacteria (Viviani, 2002). The key component involved in the generation of BL is the light emitting chemical luciferin, which is generated by a series of reactions involving the enzyme luciferase. As numerous organisms secrete luciferase and luciferin, this enzyme and molecule respectively are generally named along with the organism/species or group, for example – firefly luciferin (White et al., 1961). The enzyme luciferase catalyzes the oxidation of the luciferin to yield luminescence (Yagur-Kroll et al., 2010). There is huge variation in the reactions occurring in different organisms for the generation of luciferin. However, one of the key and common factors is the requirement of molecular oxygen and other cofactors along with luciferase, to complete the reaction. For example, the generation of firefly luciferin involves a chemical reaction involving luciferase, magnesium and adenosine triphosphate (ATP), to yield luminescent luciferin and by-products like adenosine monophosphate, CO_2_, and pyrophosphate (Marques and Esteves da Silva, 2009; Kim et al., 2015). The mechanism of this reaction is addressed in Figure 1. For generation of another luminescent molecule – photoprotein aequorin, co-factors like divalent calcium or magnesium ion are required (Brown et al., 2019). A well-known application of BL based on these reactions is the cloning of genes for the enzyme firefly luciferase and the photoprotein from the jellyfish Aequorea. This gene can be transferred into a variety of organisms including bacteria, plants, and human cells. Luminous tobacco plants and *Escherichia coli* (expression of genes of luciferases resulted in visible shades of orange, yellow and green bacterial cells) are some of the well-known examples (McCapra, 1990).

Chemiluminescent reactions have a great role in molecular and cellular based assessment due to its high sensitivity (Créton and Jaffe, 2001). For example, CL indicators are used for immunoassays, western blots, northern (nucleic acid detection) and southern blots. One of the key features of CL is its capacity to evaluate the specimen at the cellular level wherein the cells can be either live, in fixed state or hybridized with the CL probes (Roda et al., 2000a; Sala-Newby et al., 2000). CL has great potential to analyze *in vivo* systems because of the absence of the use of heat or exciting light which aids in measurements without any disturbance to the living system. This is an improvement over regular fluorescence imaging which requires high intensity emitting light and long exposure to short wavelength lights which may subsequently damage the living cells (Créton and Jaffe, 2001).

Bioluminescence and CL imaging are interesting tools for biomedical studies, clinical diagnosis, and drug development research. They have the potential to identify and analyze (quantitatively and qualitatively) enzymes, drug and drug metabolites, nucleic acids such as DNA (Qi et al., 2018), micro RNA (Ling et al., 2018), proteins (Li et al., 2015), and antigens (Mao et al., 2019) in various specimens. These specimens can be living cells in experiments, or fixed, cryo- and paraffin-embedded cells/tissues samples and sections (Roda et al., 1996, 2000b). The resolving power of the CL is capable of penetrating to the subcellular level and image the tissue section or a single cell (Roda et al., 2003a). BL and CL offer substantial advantages over other fluorescence imaging methods, which mainly includes its broad range and sensitivity in imaging samples in both micro and macro scale. In addition, the imaging is not affected by the sample matrix, because the luminescence is generated by specific set of chemicals involved in the reaction (Roda et al., 2003b).

Recently, CL intensity has been further improved by using colloidal enhancers that have enabled improvement to not only imaging and but also therapies. Colloidal enhancers can include gold (Li et al., 2008; Yan et al., 2019), silver (Chen et al., 2007; Haghighi and Bozorgzadeh, 2010), platinum (Xu and Cui, 2007), and magnetic (Yang et al., 2019) nanoparticles (NPs). For *in vivo* imaging, peroxalate nanoparticles specific are known for their high specificity and selectivity against hydrogen peroxide, and they have therefore been used for deep tissue CL imaging of the inflammatory response of mice (Lee et al., 2007). Similarly, polystyrene nanoparticles stained with squaraine catenane endoperoxide dyes, which are concurrently chemiluminescent and fluorescent, displayed significantly higher CL than fluorescence, which helped in the imaging of distribution of nanoparticles in mice (Lee et al., 2013). Similar to colloidal and inorganic NPs, enhancement to CL has been investigated with the help of quantum dots (QDs), including both metallic (Song et al., 2019) and non-metallic (Wang D. M. et al., 2019) QDs. Subsequently, enhancement effects such as chemiluminescence resonance energy transfer (CRET) has been discovered (Yao et al., 2017). CRET allows transfer of energy from a chemiluminescent donor to a fluorophore acceptor (e.g. QDs) without the need for an external excitation source, and with low background signal. Although QDs have several advantages, concerns regarding toxicity and environmental contamination have been raised (Ron, 2006; Wang et al., 2015; Li et al., 2016; Zhang et al., 2016). Chemiluminescence can also be triggered using electrochemical techniques, and this method is called electro-chemiluminescence (ECL). Upconversion nanoparticles, which are generally lanthanide- or rare earth- doped materials, have been identified and developed as ECL emitters. Unlike QDs, these particles have low toxicity, good ECL intensity, and low autofluorescence background. Detailed description of the use of upconversion nanoparticles in ECL is already available in literature (Liu et al., 2014; Gao et al., 2017; Zhai et al., 2017; Gu et al., 2019). Alternative approaches for safe enhancement of CL will be discussed below.

## Chemiluminescent Materials

Some of the most commonly used materials in CL imaging have been described in detail below.

### Luminol

Luminol is synthesized in the presence of triethylene glycol by a reaction involving cyclocondensation of 3-nitrophthalic acid with hydrazine to produce 5-nitro1,4(2H,4H)phthalazinedione. The resultant product is further reacted with sodium dithionite in the presence of heat, followed by treatment with acetic acid to produce luminol on cooling (Maynard, 1997). CL probes consisting of luminol are known to successfully detect and quantify intracellular and extracellular reactive oxygen species (ROS) produced by phagocytozing cells in the blood (Jancinová et al., 2017). Luminol and its derivatives possess an intermediate called α-hydroxyperoxide, which is derived by the oxidation of the heterocyclic ring, and this reaction depends solely on the pH of the system (Dodeigne et al., 2000). Masking or structural modification of this heterocyclic ring (Jancinová et al., 2017) results in a complete loss of these chemiluminescent properties (Dodeigne et al., 2000). Kwon et al. (2014) took advantage of this property to develop a CL chemodosimeter, in which a masking group was incorporated to prevent formation of the α-hydroxyperoxide intermediate, thereby preventing CL. When the masking group was selectively removed using a target analyte, the CL could be turned on to get a highly selective and sensitive signal (Kwon et al., 2014). CL is emitted solely due to the presence of oxygen and a strong base in an aprotic media such as DMSO, while protic solvents are capable of oxidizing luminol derivatives only with the assistance of either enzymes or mineral catalyst (Giussani et al., 2019).

Luminol has been used to generate CL via numerous techniques, for instance, by pulsed laser light or optically pumped CL. A dye absorbing red light is generated by a pulsed laser light and this light is capable of oxidizing luminol, thus generating CL (Khan et al., 2014). Luminol and its derivatives have found wide applications in diagnostic and monitoring techniques of non-immunoassay or immunoassays. Isoluminol derivatives have displayed increased efficiency and have been found to be the sole tracers used in substrate-labeled immunoassays (Dodeigne et al., 2000). Luminol has displayed a higher efficiency when its present in the free state. It has found wide applications in enzyme labeled immunoassays, detection of hydrogen peroxide, metal ions, amines, carbohydrates, vitamins, nitrate, enzymes and enzyme substrates, amino acids, cyanides and carbohydrates (Kugimiya and Fukada, 2015). Luminol has also been extensively used as a forensic tool in the form of aerosols by many police agencies in the U.S. for detecting trace blood patterns at crime scenes (Stoica et al., 2016). Luminol reacts with the reactive ROS and emits light via luminol CL (Chen et al., 2004). ROS are produced by defensive cells like macrophages and monocytes that are highly populated in a cancerous environment. Alshetaiwi et al. (2013) successfully demonstrated that luminol administration in tumorous mice allowed early stage imaging of the tumors. Inflammation produces myeloperoxidase (MPO) released by neutrophils and these superoxides react with Luminol emitting luminescence which enables investigation of different stages of inflammation (Tseng and Kung, 2013). Bedouhène et al. (2017) showed that in the presence of horseradish peroxidase, luminol-based CL can be used to detect superoxide anions and hydrogen peroxide. This method can therefore be used to detect ROS production by neutrophils, with high sensitivity. Luminol has also been incorporated within nanoparticles for CL imaging. Xu et al. (2019) recently developed a self-illuminating nanoparticle using an amphiphilic Ce6-luminol-polyethylene glycol (CLP) polymer. The Ce6 (chlorin e6), a photosensitizer, can be excited by the BL from luminol in the presence of excess ROS and myeloperoxidase. This excitation leads to generation of fluorescence and ^1^O_2_ by the Ce6 via bioluminescence resonance energy transfer (BRET), and can be used for detecting inflammation and for tumor photodynamic therapy (PDT) (Xu et al., 2019).

### Coelenterazine

Coelenterazine is derived from a protein called coelenterate, which has been synthesized by several methods described elsewhere (Dodeigne et al., 2000). This compound possesses a superoxide anion in its structure, which is responsible for causing coelenterazine to give out CL. Unlike luminol, coelenterazine does not require any catalyst to trigger CL (Silva et al., 2012). CLA (2-methyl-6-phenyl-3,7-dihydroimidazo[1,2-a]pyrazin-3-one) and specifically MCLA probe (2-methyl-6-(4-methoxyphenyl)-3,7-dihydroimidazo[1,2-a]pyrazin-3-one) which is more efficient are some of the several coelenterazine analogs that have been prepared and used (Dubuisson et al., 2005; Wang et al., 2012; Diaz et al., 2018). In contrast to luminol, MCLA is cell impermeable, and is therefore useful for detection of superoxides outside the cell (Diaz et al., 2018). Besides being widely employed for monitoring of superoxide, coelenterazine and its analogs have found wide applications as prosthetic groups of various photoproteins like mnemiopsin, aequorin, phialidin obelin, and beroverin. Of all the above mentioned photoproteins, aequorin is widely used for measuring intracellular calcium and in immunoassay applications (Nguyen et al., 2018; Feno et al., 2019). Coelenterazine has been used often in cancer imaging. CL produced by coelenterazine are used to estimate the elevated levels of ROS that are produced by cancer cells (Bronsart et al., 2016a). Coelenterazine has also been used to detect and image chronic inflammation associated with conditions like inflammatory bowel disease, as it produces CL upon reaction with ROS associated with inflammation (Bronsart et al., 2016b). Bronsart et al. (2016a) were able to detect chemiluminecence *in vivo* at 3 and 6 days after intravenous administration of coelenterazine in tumorous mice. Wang Y. et al. (2002) successfully combined colenterazine with a fusion gene construct which enabled real-time imaging of gene expression both in cell culture and animal models.

### Peroxyoxalic Acid and Their Derivatives

Peroxyoxalate CL is achieved in the presence of a base catalyst and an appropriate fluorophore by combining hydrogen peroxide with oxalate ester (Dodeigne et al., 2000). Peroxyoxalic acid and their derivatives undergo oxidation in the presence of hydrogen peroxide producing high-energy intermediates which is dioxetanedione. Peroxyoxalate and its derivatives have found wide applications in determining selective fluorophores especially after separation by high performance liquid chromatography (Huertas-Pérez et al., 2016). However, as compared to the previously mentioned compounds, fluorescence is not emitted by the high-energy intermediate itself. Light emission is produced by energy transfer to a fluorescer, which gets excited in a S1 state (Smellie et al., 2017). The oxalate compound and the fluorescent sensitizer can be chosen independently. However, as compared to other chemiluminescent producing compounds, the efficiency of the peroxyoxalic acid and their derivatives are reportedly low. The efficiency of this fluorescent material is higher in organic solvents as compared to aqueous solvent mixtures (Dodeigne et al., 2000).

Another limitation is the observance of high background in peroxyoxalate CL which is produced due to the blending of hydrogen peroxide and peroxyoxalate (Cepas et al., 1995; Romanyuk et al., 2017). This background emission can be suppressed by addition of continuous reagent like *bis*(2,4,6-trichlorophenyl)oxa-late (TCPO)-hydrogen peroxide system (Niu et al., 2006). The TCPO system have been used to detect the protein labeled 2-methoxy-2,4-diphenyl-3(2H)-furanone (MDPF) (Salerno and Daban, 2003). Another disadvantage of this material is the poor stability of the compound in water or aqueous solutions since partial water hydrolysis results in the decomposition by decarboxylation and decarbonylation, limiting its application in diagnostics (Delafresnaye et al., 2019). Peroxyoxalate chemiluminescence (POCL) has also found wide applications in detecting hydrogen peroxide-forming enzymes namely cholesterol oxidase, uricase, xanthine oxidase, glucose oxidase, and choline oxidase (Nozaki and Kawamoto, 2003). It has also been used to eliminate tumor cells, where the CL can be absorbed by photosensitizers accumulating within the tumor, resulting in singlet oxygen generation and subsequent cell death (Romanyuk et al., 2017). POCL-containing nanoparticles have also been studied, which are sensitive to endogenous hydrogen peroxide and can be used to study inflammation, where overproduction of hydrogen peroxide is expected (Lee et al., 2007). Peroxalate loaded nanoparticles injected into the peritoneal cavity demonstrated high specificity and selective imaging of hydrogen peroxide-related inflammatory diseases (Lee et al., 2007). POCL has been used in literature frequently in nanoparticle and hydrogel preparations. Li et al. (2019) developed a POCL nanoparticle – glucose oxidase-doped alginate hydrogel, in order to determine glucose levels in the tumor periphery to study tumor metabolism. Following glucose permeation into the system, it will be oxidized by glucose oxidase to produce H_2_O_2_, which will be detected by peroxyoxalate. Following subcutaneous injection of the solution into CT-26 tumor bearing mice, the gel allowed localization of the nanoparticles to provide a high signal-to-noise ratio at the tumor site (Li et al., 2019).

### Acridinium Esters

Acridinium esters possess high quantum yields that can be detected in the attomole range (Weeks et al., 1983; Natrajan et al., 2010). In comparison with other materials, simple chemical triggers of acridinium derivatives display quick light emission with their minute size permitting easy labeling protocols of nucleic acids and proteins. Acridinium phenyl esters display greater luminescence than simple alkyl esters (Natrajan et al., 2010). Unlike the other chemiluminescent materials, acridinium do not require a catalyst to produce CL. Hydrogen peroxide and a strong base are sufficient to cause them to produce chemiluminescence (Dodeigne et al., 2000). Another advantage is their ability to exhibit faster light emission with simple chemical triggers (Natrajan et al., 2010). The main disadvantage of this chemiluminescent material is its instability in aqueous medium as the ester bond that is present between the acridinium ring and the phenol undergoes hydrolysis (Brown et al., 2009; Natrajan et al., 2010).

Despite this limitation, acridinium derivatives have found wide applications in immunoassays. Acridinium ester has been successfully used to perform ultrasensitive immunoassays of various proteins and antibodies. tumor markers (a-fetoprotein), thyroid stimulating hormone (TSH) and immunoglobulins (Ma et al., 2017; Min et al., 2018; Chen et al., 2019). Acridinium ester is also able to successfully label strands of DNA to produce DNA probes to emit CL (Komori et al., 2019). In a study, wild-type p53 was immobilized on the surface of gold-functionalized magnetic nanoparticles. 2′,6′-dimethylcarbonylphenyl-10-sulfopropylacridinium-9-carboxylate 4′-NHS ester was mixed with the complementary sequence of wild-type p53. The two samples were mixed and the gold-conjugated magnetic nanoparticles were subsequently separated. CL imaging showed ultrahigh sensitivity and selectivity in detecting the p53 tumor suppressor gene up to a limit of 0.001 ng/mL (Wang L. et al., 2019). Several other applications include estimating thermodynamic affinities of oligonucleotide probes that are bound to simple synthetic as well as complex biological targets and hybridization rate constants (Créton and Jaffe, 2001; Nakazono et al., 2019).

### FCLA (3,7-Dihydro-6-[4-[2-[N0 -(5- Fluoresceinyl)thioureido]-Ethoxy]phenyl]- 2-Methylimidazo[1,2- a]pyrazin-3-One Sodium Salt)

FCLA is a highly efficient water soluble chemiluminescent agent (He et al., 2002a). FCLA is an analog belonging to *Cypridina* luciferin that efficiently reacts with superoxide anion (^O_2^–^) or singlet oxygen (^1^O_2_) that emits luminescence via a dioxytane intermediate (He et al., 2002a). *OH*^−^ +*NaOCl*+*H*_2_*O*_2_, a typical reaction system is involved in generating a singlet oxygen which produces emission at about 532 nm (Wang Y. et al., 2002). Researchers have developed a novel method to diagnose superficial tumors by photodynamic diagnosis mediated by CL probe containing FCLA (Wang J. et al., 2002). Wei et al. (2011) utilized FCLA CL to monitor tumor necrosis in response to photodynamic therapy. First the FCLA was injected subcutaneously in mice, and light irradiation was provided after a 1 h. A near linear relationship was observed between the extent of damage from PDT, and the CL (Wei et al., 2011). FCLA CL was also used recently to detect ROS generation following DNA duplex-based photodynamic therapy against retinoblastoma (Wei et al., 2016).

There are several examples in literature of coupling chemiluminescent probes with enhancers such as US. Due to US’ ability to penetrate deep within the tissues and remain targeted to a small region, undesirable side effects can be minimized and greater spatial information of the CL molecules can be obtained (He et al., 2002b; Kobayashi and Iwasa, 2018). Ultrasonic irradiation of water results in acoustic cavitation producing ^•^OH and ^•^H, which form active oxygen species. These oxygen species react with FCLA producing CL (He et al., 2002b). He and colleagues observed that, when a sonosensitizer (hematoporphyrin derivative) accumulating in tumor tissues was exposed to ultrasound *in vivo*, FCLA reacted with the resulting active oxygen species to emit CL. This CL was stronger from the tumor region in comparison from other regions. He reported that the application of US increased the intensity of chemiluminescence emitted by FCLA (He et al., 2002b; Wei et al., 2016) and it resulted in excellent signal-to-noise ration ratio of a sonoluminescence image of tumorous mice on subcutaneous injection of FCLA solution (He et al., 2002a).

## Second Generation of Chemiluminescence: Ultrasound-Enhanced Chemiluminescence

There are many advantages to using US and CL simultaneously. Since both US and CL are imaging techniques, they can be used for dual imaging in order to accurately visualize the tissue of interest. In addition, US can enhance the intensity of CL by reducing light scattering while increasing spatial resolution. Both of these approaches are described below.

### Combination of US and CL for Dual Imaging

**FIGURE 2:**
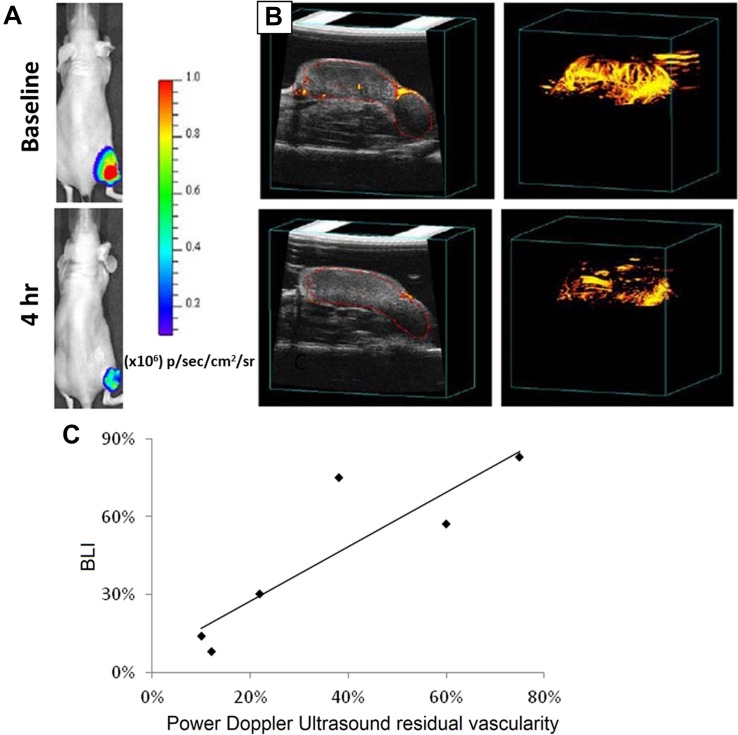
Images captured for tumor vasculature disruption before (top) and 4 h after (bottom) injection of arsenic trioxide (8 mg/kg) using **(A)** Bioluminescent and **(B)** US techniques. **(C)** Graph comparing US and BLI in tumors as fractional signal versus baseline. Nude mice bearing MCF7-mCherry-luciferase tumors were used. Reprint (Alhasan et al., 2012) under open-access terms of Creative Commons Attribution License.

In an *in vivo* study by Alhasan et al. (2012) on tumor (luciferase-transfected PC3 cancer cell lines) bearing nude mice, *D*-luciferin-based bioluminescent imaging (BLI), fluorescence imaging and doppler ultrasound imaging techniques were simultaneously and independently conducted. Both BLI and US were able to correctly indicate time-dependent percent reduction in tumor blood flow following the injection of arsenic trioxide (ATO) – a model vascular disrupting agent (Figures 2A,B), and a correlation was obtained (Figure 2C) with *R*^2^ > 0.77 (Alhasan et al., 2012). On the other hand, fluorescence imaging did not show any changes in the first 24 h following ATO administration. Jung et al. (2018) developed curcumin-containing antioxidant vanillyl alcohol-incorporated copolyoxalate (PVAX) nanoparticles, which can be simultaneously used for anti-cancer therapy, peroxalate CL, and amplification of ultrasound signals through generation of H_2_O_2_ triggered CO_2_ bubbles at ischemic sites. More recently, Liu et al. (2019) used nanobubbles doped with luminol, 1,1′-Dioctadecyl-3,3,3′,3′-tetramethylindocarbocyanine perchlorate (DiI) and 1,1′-dioctadecyl-3,3,3′,3′-tetramethylindodicarbocyanine perchlorate (DiD) dyes for dual BLI and US imaging. The luminol could detect myeloperoxidase activity in areas of inflammation and emit a blue light. By integrating BRET and fluorescence resonance energy transfer (FRET) using the DiI-DiD, the light can be shifted into red light. This method was used in combination with ultrasound imaging to get more information on anatomical structure and vasculature (Liu et al., 2019). This shows that US and BLI are useful tools that may be used independently or simultaneously to obtain important vasculature-related and anatomical information while providing therapy. However, in majority of the cases described in recent literature, US has been chiefly used as a tool for enhancing CL.

### Mechanism of UECL

Although CL is widely used in tissue imaging, a chief concern raised in literature is that the scattering light increases noises of the detection. The increase in noises could be explained using the following redox reaction, similar to the Fenton reaction (McMurray and Wilson, 1999):

      
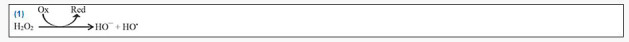


From Eq. (1), it could be interfered that, with the ubiquitous appearance of oxidizing agents in tissues, free HO^•^ are also commonly produced within tissues. Subsequently, to increase signal to noise ratios, one could reduce the background signals by locally increasing production of H_2_O_2_ or free HO^•^ of the target tissue while reducing those amounts in the nearby medium.

The possibility of using US to enhance sensitivity of CL was first discussed two decades ago in a non-tissue mechanistic study by McMurray and Wilson (1999). Although their system was non-tissue, it was proven that the intensity of sonochemiluminescence, I_SCL_, was linearly increased with the increase of US power up to 100 W. The study was conducted at 10^–3^ M luminol and 10^–4^ M H_2_O_2_. McMurray proposed that, at the air-liquid interface of the cavitation bubbles, water and oxygen molecules were freed and more local free radicals were created.

(2) $${\text{H}}_{2}\,\text{O}\to {\text{HO}}^{-}+{\mathrm{HO}}^{•}$$

(3) $${\text{O}}_{2}\to 2\,{\text{O}}^{•}$$

**FIGURE 3:**
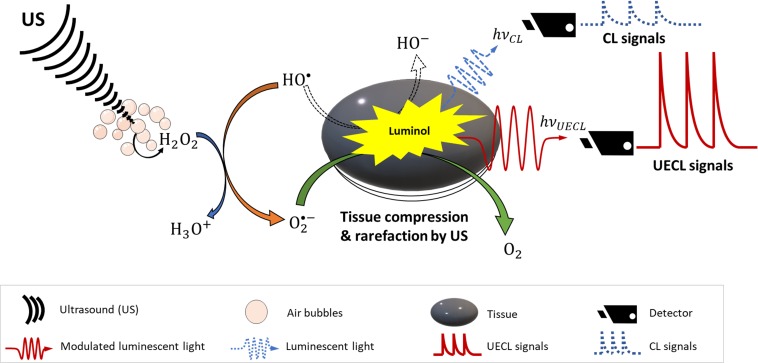
Schematic design of ultrasound-enhanced chemiluminescence. Dash arrow and waves are mechanism and signals without ultrasound enhancement; solid arrows and waves are mechanism and signals with ultrasound enhancement.

In term of mechanical and physical properties of tissues, it has been shown that focused ultrasound (FUS) creates periodic compression and rarefaction of tissues, which changes refractive indices of tissues locally and allows less optical absorption and scattering (Li and Wang, 2004; Murray et al., 2004). Laser light can also be modulated with frequency of US. It has been discussed in literature that tissues oscillate with US frequency that subsequently produce harmonic interference to laser light (Li and Wang, 2004; Murray et al., 2004; Jarrett et al., 2014). Meanwhile, a phenomenon called photon–phonon interaction (Kempe et al., 1997; Jarrett et al., 2014) modulates the frequency of the transmitted laser light. Consequently, laser light is modulated to transverse deeper into tissues with less reflection. A detailed schematic of the mechanism of UECL is shown in Figure 3.

### Effects of Ultrasound on Chemiluminescent Signals

Since observing that chemiluminescent signals can be modulated by US, there have been more research conducted to understand this concept. A detail mechanism was proposed (McMurray and Wilson, 1999), who suggested that I_SCL_ was correlated to free radical HO^•^ concentration, which was confirmed to be linearly proportional to γ-ray pulse radiolytic dose or US power. More interestingly, the authors reported that effective distances were strongly dependent on the alignment of US waves. This means that the more focused the US delivered, the more aligned or less scattered the luminescent light was, and therefore the higher the resolution of the CL signal recorded.

**FIGURE 4:**
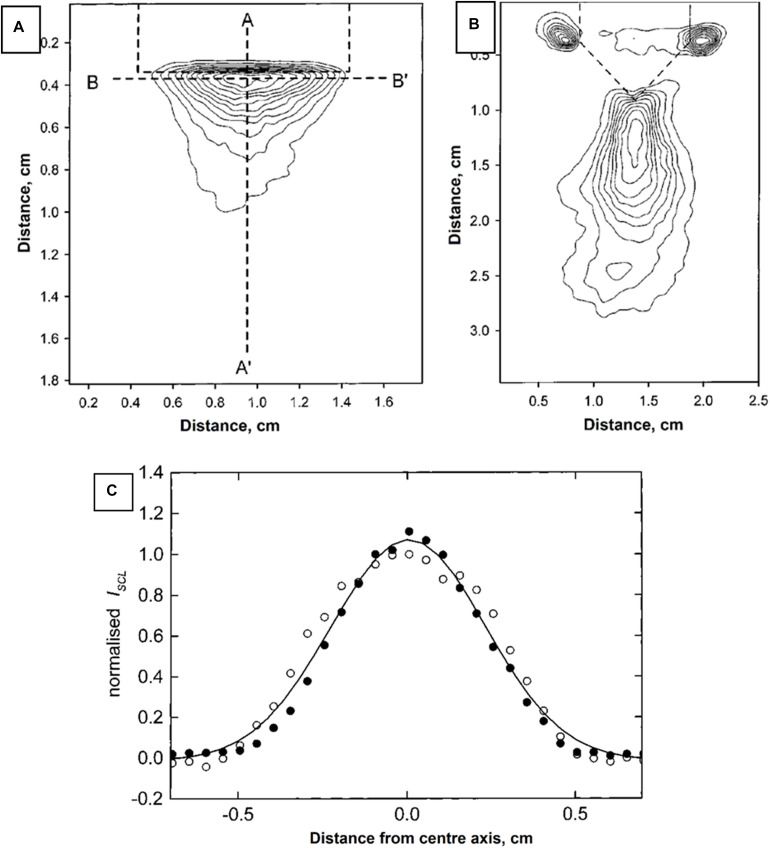
US-dependent luminol CL when using **(A)** flat and **(B)** wedge-ended sonoprobe tips. Bell-shape distribution **(C)** indicated highly penetration of US in supporting CL. Reproduced with permission from McMurray and Wilson (1999).

From Figures 4A,B, it can be clearly observed that, with increased focus of US, the CL signals were also correspondingly increased. This result indicates FUS can significantly enhance sensitivity of CL (Figure 4C) by enhancing the distance that the CL laser can travel (Figure 3). The mechanism behind this is unclear; however, it might be due to the fact that the temperature of focal points was increased locally by the US, and it has been noted elsewhere in literature that increases in temperature could increase sensitivity of CL. Aslan et al. (2006) has previously reported that all red, blue and green CLs were increased up to 75-fold through heating although in this case the heat was introduced by microwaves.

**FIGURE 5:**
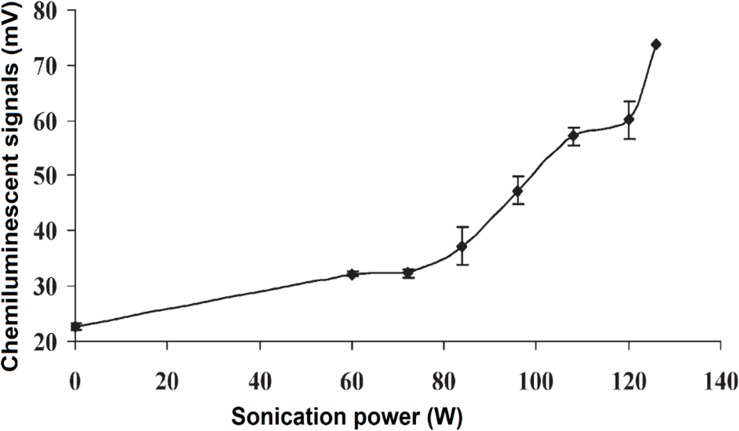
CL intensity was increased with the increase of ultrasonication power. Reproduced from Greenway et al. (2006) with permission from The Royal Society of Chemistry.

In correlation with the findings by McMurray and Wilson (1999) and Greenway et al. (2006) studied the effects of US power and the distance of US probe from the sample, on CL signals (Figure 5). The signals were reported to be significantly enhanced with the distance of 2–8 mm, and was dependent on the US power (between 60 and 126 W) (Greenway et al., 2006). In agreement with McMurray and Wilson (1999) and Greenway et al. (2006) suggested a mechanism where ultrasonication produces H_2_O_2_ that subsequently stabilized the short-lived free radicals HO^•^ as below:

(4) $${\text{HO}}^{•}+H_{2}\,{\text{O}}_{2}\to {\text{O}}_{2}^{•-}+H_{3}\,{\text{O}}^{+}$$

The stabilized ${\text{O}}_{2}^{•-}$ would then react with luminol and produce increased signals. This mechanism is supported by the fact that CL intensity has been reported to increase in alkaline solutions (McMurray and Wilson, 1999; Miyoshi et al., 2001) where reaction (4) is accelerated to the right and produce more ${\text{O}}_{2}^{•-}$. Recently, similar mechanism of ${\text{O}}_{2}^{•-}$ enhancing CL has been proposed by Chen et al. (2014), where quantum dots utilized ${\text{O}}_{2}^{•-}$ to enhance the signals of CL. For FUS, it is able to control the power and focal point, thus it concretes for the potential of US in enhancing sensitivity of CL.

**FIGURE 6:**
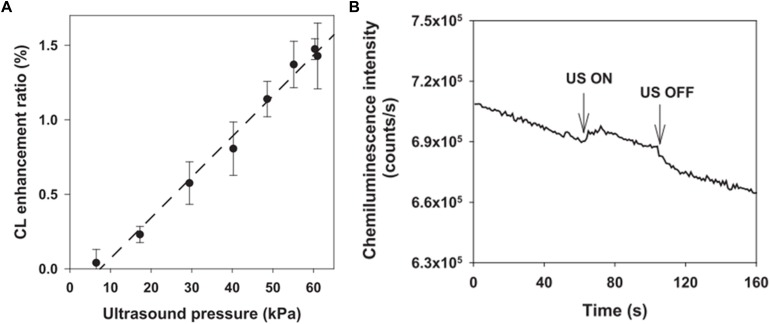
**(A)** CL intensity was increased with the increase of FUS pressure. **(B)** Changes in CL signals in the presence and absence of FUS triggers. Reproduced from Kobayashi et al. (2015) with permission from Applied Physics Letters.

Recently, researchers have been studying UECLs on tissue environment mimics. Huynh et al. (2013) studied FUS enhancing CLs through a gel tissue phantom as a scattering medium. Using 1 MHz US transducer and creating cavitation pressure 0.42 MPa, the authors detected luminescent objects at depth of 7 mm with 10 times more sensitivity than traditional luminescent methods (Huynh et al., 2013). The resolution was reported at 3 mm. Huynh et al. (2013) also indicated that the application of microbubbles, which were FUS contrast agents, could enhance CL. In agreement with this research, Kobayashi et al. (2015) also reported that the sensitivity of POCL was increased along with the increase of inner pressure up to 6 MPa created from FUS. A clear increase and decrease in CL signals were recorded with on and off stimulation of FUS (Figure 6).

We can see from the research reviewed above that theoretical studies and lab bench experiments have proven that US, especially FUS, does have beneficial effects on increasing the sensitivity of CL. However, there is a need for evidence of efficacy on tissue-based systems. For this, scientists have mimicked tissue microenvironments using turbid medium and *ex vivo* tissues for their studies.

### FUS-CL in Turbid Microenvironments

**FIGURE 7:**
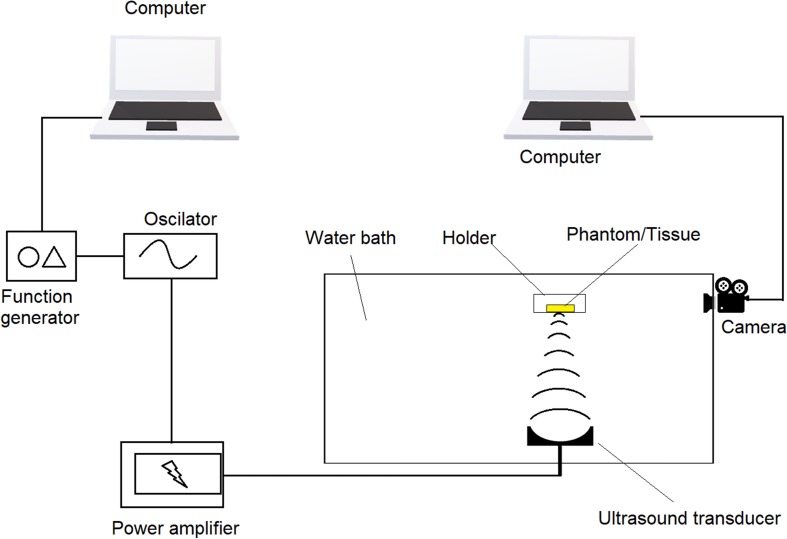
Schematic design of an imaging system utilizing Ultrasound-enhanced chemiluminescence.

In order to mimic tissue environments, agarose phantom is usually used as it has similar scattering coefficient as that of native tissues following Monte Carlo model (Wang et al., 1995). Since native tissues may vary from species to species, phantoms typically have a scattering coefficient from 1 to 80 cm^–1^ (Kobayashi et al., 2016; Ahmad et al., 2017; Zhu et al., 2018). Through phantoms, transparent silicon tubes were run at different depths. CL solutions passing through the silicon tubes were recorded with or without stimulation of US that is focused at the center of the tubes. Minimum distance between two tubes that generate non-identical signals are considered as resolution. Schematic design of UECL imaging system is illustrated in Figure 7.

Using FUS at different stimulation levels, authors not only observed 10 times higher signal-to-noise ratio (Zhu et al., 2018) for 1064 nm luminescent laser but also increased sensitivity or better spatial resolution. The best resolution was reported at 2 mm (Ahmad et al., 2017; Klein et al., 2018) using 640 nm laser or ^18^F lasers. The deepest penetration were obtained at 25–30 mm (Kobayashi et al., 2016) recorded on agarose phantom using POCL system. Interestingly, focused US at a low power could enhance POCL using indocyanine green (ICG) as the fluorophore (Kobayashi et al., 2016). In most cases in literature, we can see that the more powerful the FUS, the greater the penetration and resolution. For instance, once FUS was increased from 1 to 2 MHz, signals were 10 times stronger as normalized to background (Klein et al., 2018; Zhu et al., 2018) while increasing FUS from 2 to 3 MHz allowed fourfold deeper penetration and 33% better resolution (Ahmad et al., 2017; Zhu et al., 2018).

### FUS-CL in *ex vivo* Studies

**FIGURE 8:**
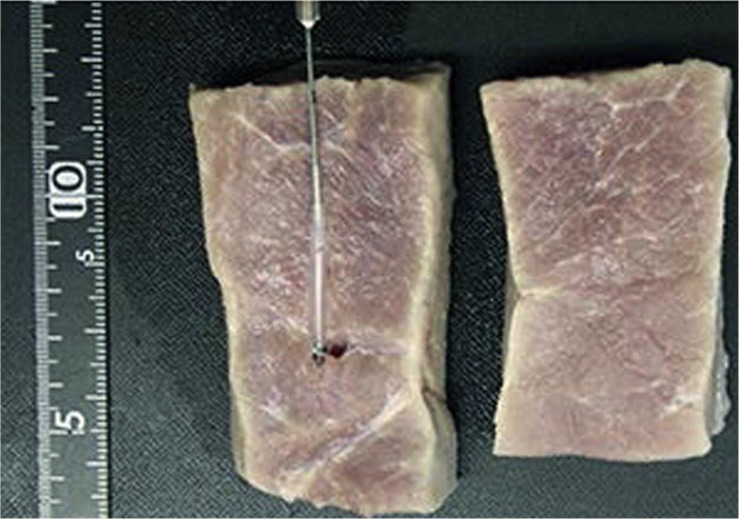
**(A)** Images of an incised porcine muscle tissue in which a CL target capsule consisting of a 0.5 mm silicone tube, was embedded. **(B)** Two-dimensional tomographic image obtained with the slab of porcine muscle. The tissue, measuring 50 mm (X) × 50 mm (Y), had a CL target embedded within it to a depth of 25 mm. Reproduced with permission from Kobayashi et al. (2016).

FUS-enhanced CL have also been studied *ex vivo* on dissected tissues and outcomes have been promising. Ahmad et al. (2017) observed clear peaks of two chemiluminescent sources (encapsulated inside plastic tubes) placed at 10 mm distance in chicken breast tissue at depth of 20 mm. Meanwhile, the experiments of Kobayashi et al. (2016) were even more interesting that they reached resoluble signals at a depth of 25 mm (Figure 8) in porcine tissues. On the other hand, Dawood (2016) demonstrated that greater penetration depth of lasers [637, 808, or 1064 nm Nd:YAG (Neodymium-dopped Yttrium-Aluminum-Garnet (Julian, 2016) laser probes] was achieved through 10 mm bovine tissue with help of FUS (Dawood, 2016) for the fact output intensity was increased by 35–45% while attenuation was decreased 3–10%. The more powerful the FUS, the greater the penetration depth of the laser light in the tissue, without having to increase the laser power (Dawood, 2016). This clearly indicates the potential of US to enhance CL by allowing greater penetration of laser through decreasing their attenuation coefficient while passing through the tissues. To summarize, we can see from literature that US at high power (typically 3 MHz and above or 5 W/cm^2^) can enhance CL signals and can enable imaging of tissues as deep as 30 mm at a resolution as high as 2 mm. Summary of FUS enhanced CL is provided in Table 2 above.

**TABLE 2:** Summary of *ex vivo* and *in vivo* applications of UECL.

**Ultrasound type**	**Power**	**CL probe**	**Increased signal^ξ^ (folds)**	**Tested environment**	**Depth^*ξ*^**	**Spatial resolution^λ^**	**Reference**
FUS	2 MHz	Nd:YVO4 (Neodymium-doped yttrium orthovanadate) laser with 1064 nm wavelength; Embedding aluminum foil as target	10 less signal-to-noise	Agarose phantom from 20% w/v intralipid. Scattering coefficient 1–4 cm^–1^	5 mm	3 mm	Zhu et al., 2018
FUS	3.5 MHz; 1 MPa	640 nm emitting CL probe	∼7-folds	Agar phantom from agar and polystyrene microspheres. Scattering coefficient 80 cm^–1^	∼20 mm	2 mm	Ahmad et al., 2017
FUS	1 MHz; 10 MPa	^18^F, tracking Cerenkov photons	N/A	Agarose phantom containing 250 μM voxels. Scattering coefficient 10 cm^–1^	5 mm	2 vs. 6 mm without US	Klein et al., 2018
Low power FUS	< 0.14 W/cm^2^	POCL system		Agarose phantom from 10% w/v intralipid, 20% w/v glycerol and 2% w/v agarose; Scattering coefficient 15 cm^–1^	25–30 mm	6 mm	Kobayashi et al., 2016
Low power FUS	<0.14 W/cm^2^	POCL system	∼1.5	Porcine muscle	25 mm		Kobayashi et al., 2016
FUS	3.5 MHz; 1 MPa	640 nm emitting CL probe	∼9–11-folds	Chicken breast	∼20 mm	10 mm	Ahmad et al., 2017
FUS	3.3 MHz;	637 nm diode, 808 nm diode, and 1064 nm	∼1.35–1.45-fold in power output;	Bovine muscles	10 mm		Dawood, 2016
	5 W/cm^2^	Nd:YAG lasers	∼7–10% decrease in attenuation (637 and 808 nm lasers); ∼3% decrease in attenuation (Nd:YAG lasers)				

*ξ: larger is better; λ: smaller is better.*

## Conclusion and Future Outlook

The significant enhancement of CL with the help of US has been proven by theoretical demonstration, using tissue mimics, and by *ex vivo* studies. The enhanced spatial information and reduced light scattering observed when combining ultrasound with CL is promising for deeper imaging of biological tissues. Among all the mechanisms being studied for CL enhancement using US, the use of US modulated laser light has shown most promise for practical medical imaging. The more focused and powerful the US, the deeper the penetration, and better the resolution CL signals that is obtained. US at 3 MHz or 5 W/cm^2^ and above has given the most enhancement to CL according to literature, as described above. At this setting, tissues have been imaged up to a depth of 30 mm with a good resolution of 2 mm. Additionally, US also has the advantage of providing computational data for data processing, and this may also advance the UECL further (Pei and Wei, 2019).

There are, however, a few drawbacks to this technology. A major cause of concern is the local heat generated by US. The more the focus, the greater the heat generated. Although local heat generated by US enhances intensity and resolution of CL images, generation of heat beyond the endurance of cells and can lead to tissue damage. According to a review of previous literature by Yarmolenko et al. (2011), the threshold damage temperature is 42°C, and cumulative equivalent minutes at 43°C (CEM_43_) of more than 1 usually causes damage to tissues. To avoid the risks of overheating the tissues under consideration, the exposure duration should be optimized. For example, in a research by Pei et al. (2014), a short HIFU exposure of 0.3 s or 300 ms limited the temperature to below 43°C. According to their calculations, the CEM_43_ was 0.0013 (Pei et al., 2014). Since primary cells in tissues are vulnerable to heat, and focused UECL is limited in its ability to control local heat, there needs to be detailed investigation into methods to overcome this limitation of FUS so that it can be used broadly without tissue damage.

With the emerging interest in FUS studies and the development of sophisticated technology, UECL is expected to play a greater role in tissue and molecular imaging in the coming years. In the future, we can expect to see more sophisticate formulations involving drug-loaded microbubbles tagged with CL probes, for theranostic applications. The microbubbles can be used not only for imaging but also for delivering therapeutics for treatment. This would mean that under stimulation of US, laser light can be modulated to penetrate deeper into tissues while microbubbles will deliver the therapeutics in response to the US stimuli. In return, the chemiluminescent images may give *in situ* feedback about the delivery process. The electroluminescence upconversion particles, which are already gaining significant attention in recent years, can also be combined with ultrasound to further enhance their properties for imaging. These particles possess good biocompatibility but low toxicity, high photostability and low photobleaching (Jin et al., 2018; Gu et al., 2019), and they are able to convert near infra-red (NIR) at deep penetration up to 10 mm (Yang et al., 2012; Jin et al., 2018; Gu et al., 2019) into visible radiation. Their photostability plus penetration ability and the aforementioned UECL ability to modulate NIR lasers *ex vivo* could possibly return in synergistic effects. Additionally, a label-free imaging technique called non-linear optical microscopy (NLOM), has been studied for both precision and safety advantages. The principle of label-free non-linear optical microscopy is based on two-photon excited fluorescence (TPEF) from cofactors nicotinamide adenine dinucleotide (NADH) and flavin adenine dinucleotide (FAD^+^) that provides high-resolution cellular redox imaging (Hou et al., 2018). More interestingly, this technique shares the same redox reactions to CL and UECL, therefore the two techniques could possibly image the tissues simultaneously and complement each other. Another technique that could also complement CL and UECL for imaging of shallow tissues is surface-enhanced Raman scattering (SERS). It has been reported that SERS have been successfully applied in small animal *in vivo* diagnostic and cancer detection (Henry et al., 2016). When using nanotags as enhancers, SERS alone or in combination with Spatially offset Raman Spectroscopy (SESOR), could be used to image at different depths [∼5 mm with SERS (Stone et al., 2010) and 45–50 mm with SESOR (Stone et al., 2011)] on porcine tissues. This combination is therefore expected to provide complementary information to make the imaging more comprehensive.

High intensity focused US, or HIFU, is also attracting more interest from researchers and we can expect to see more research into the use of HIFU combined with CL in the future. The development of this technology allows scientists to have greater control on the focus and localization, besides controlling the heat generated. Several versions of HIFU, namely ultrasound-guided and MRI-guided HIFU, have been tested preclinically and in pilot studies, for breast cancer, liver cancer, pancreatic cancer (Maloney and Hwang, 2015) and prostate cancer (van Velthoven et al., 2016). MRI-guided HIFU is clinically approved in the European Union for palliative treatment of bone lesions (Maloney and Hwang, 2015). The UECL technique by HIFU therefore has high translational potential. It will significantly enhance physiological imaging of living organisms by providing high resolution images, which will aid in providing accurate diagnosis and therapy in the future.

## Author Contributions

DL prepared the outline, wrote and edited the manuscript, and prepared figures and tables. DD and AG wrote the manuscript, and prepared figures and tables. JM edited and revised the manuscript. All authors approved the manuscript for publication.

## Conflict of Interest

The authors declare that the research was conducted in the absence of any commercial or financial relationships that could be construed as a potential conflict of interest.
